# Predictors of Stigma and Health-Related Quality of Life Among People Living with HIV in Northern Thailand

**DOI:** 10.1089/apc.2022.0035

**Published:** 2022-05-03

**Authors:** Ajaree Rayanakorn, Parichat Ong-artborirak, Zanfina Ademi, Suwat Chariyalertsak

**Affiliations:** ^1^ Faculty of Public Health, Chiang Mai University, Chiang Mai, Thailand.; ^2^ School of Pharmacy, Monash University Malaysia, Bandar Sunway, Malaysia.; ^3^ Epidemiology Research Group of Infectious Disease (ERGID), Chiang Mai University, Chiang Mai, Thailand.; ^4^ Centre for Medicine Use and Safety, Faculty of Pharmacy and Pharmaceutical Sciences, Monash University, Melbourne, Australia.; ^5^ School of Public Health and Preventive Medicine, Monash University, Melbourne, Australia.

**Keywords:** HIV-related stigma, quality of life, PLWHIV, HIV, Thailand

## Abstract

HIV-related stigma and discrimination have been a significant barrier to accessing health care, hence contributing to poor health outcomes. This study aimed to investigate factors associated with HIV-related stigma and discrimination and health-related quality of life (HRQoL) among people living with HIV in rural Thailand setting. A cross-sectional convenience sample of 161 HIV-positive Thai patients was recruited from a single rural district hospital using a self-administered questionnaire entailing sociodemographic information, the 12-item stigma scale, the Medical Outcomes Study HIV Health Survey (MOS-HIV), and the EuroQoL 5-Dimension 5-Level (EQ-5D-5L). Linear regression and the multi-variable analyses were used to investigate factors associated with stigma and HRQoL, whereas the correlations between stigma and quality of life variables were tested by Pearson correlations. Being married and duration of antiretroviral therapy were negatively correlated with HIV stigma, while increased age was inversely associated with HRQoL. Being employed and having sufficient money for living contributed positively to predict HRQoL. HIV stigma was negatively associated with mental health summary (MHS) and visual analog scale (VAS) score, whereas duration diagnosed with HIV and the use of two nucleoside reverse transcriptase inhibitors in combination with protease inhibitor regimen were negative factors associated with VAS and health utility, respectively. The findings confirm complex and inseparable associations of factors relating to HIV-related stigma and HRQoL. The development of effective interventions tailored at individual level is warranted to address this gap.

## Introduction

Since the first HIV/AIDS patient reported in Thailand in 1984,^1^ HIV has been one of the major public health challenges. In 2020, it has been estimated that there were over 500,000 people living with HIV (PLWHIV), 12,000 AIDS-related deaths, and 6600 new adult cases, making Thailand still one of the countries with highest HIV prevalence in Southeast Asia.^2^

Antiretroviral (ARV) therapy (ART) has demonstrated a positive impact in improving health and quality of life among PLWHIV, as well as reducing the disease transmission.^3–5^ The access to effective medication has transformed HIV as a lethal disease into a chronic condition,^6,7^ which has led to the focus shifting from prolonging life to long-term care and better health outcomes. In Thailand, ART has been included into the universal health coverage (UHC)'s benefit package since 2005, resulting in significantly decreased HIV prevalence rate to 1% and tremendous gains toward the 90-90-90 target.^8^

However, there has been a relatively modest proportion of virological suppression among those on ART with lower rates in certain groups, including men who have sex with men (MSM), transgender women (TGW), sex workers (SWs), and people who inject drugs.^8,9^ This stable trend and the gradual reduction rate of HIV infection in recent years are probably due to limited health care access to preventive measures.^8^

Despite several decades into the global epidemic, HIV remains one of the most stigmatized diseases globally.^10^ The perception that HIV is concentrated among key populations involving homosexuality, prostitution, and drug abuse is even compounded and worsen HIV-related stigmatization.^10^ A survey among 1557 health workers delivering HIV services in South Africa and Zambia showed higher levels of negative attitudes toward key populations, particularly among women who sell sex and MSM compared to PLWHIV.^11^ Stigmatization and discrimination toward PLWHIV are common and have been key barriers to accessing health care services and controlling the spread of infection.^12,13^

According to Thailand's national survey conducted in 2017, 1 out of 10 PLWHIV experienced stigma and discrimination in health care setting, whereas one-third reported avoiding health facility visit due to internalized stigma.^14^ Also, a national prospective cohort study among women living with HIV in the United States suggested a significant association between higher levels of experienced and anticipated HIV stigma in health care settings and lower levels of trust among health care providers.^15^ These have inadvertently resulted in depression and poorer health status and quality of life.^10,16,17^

The three types of HIV-related stigma include self-perceived stigma, internalized stigma, and experienced stigma,^10^ which commonly occur in three areas: social and community area, medical and health work area, and self-perceived stigma or personal perception,^18^ of which self-perceived stigma was found to more negatively impact overall well-being among PLWHIVs than external discrimination.^19^ However, research on HIV-related stigma and discrimination has been mainly focused on health care providers' and public's perspective, whereas there is paucity of research on discrimination under PLWHIV's perspective, who are the discrimination objects.^18^

This cross-sectional study aimed to examine the predictors associated with HIV-related stigma and discrimination, the status of stigmatization, and health-related quality of life (HRQoL) under the perspective of PLWHIV in Phrao district, Chiang Mai, Thailand. The findings would provide a better understanding of the relationship regarding factors influencing HIV-perceived stigmatization and HRQoL, which would be useful in designing effective intervention strategies to reduce HIV-related stigmatization and improve quality of life among PLWHIV.

## Methods

### Study design

A self-administered questionnaire to study HIV-related stigma and discrimination and HRQoL was used for data collection. The questionnaire comprised three sections. The first section contains sociodemographic information, including age, sex, educational level, occupation, marital status, years after HIV diagnosis, ART use and duration, CD4 cell counts, the number of days missed from work, and productivity impact during the recent 3 months. The second section entails the abbreviated 12-item stigma scale, the short version of Berger et al.'s 40-item HIV stigma scale, one of the most comprehensive and commonly used instruments for PLWHIV,^20^ which was previously translated and validated in Thai population by Rongkavilit et al. (Cronbach's *α* = 0.75).^21^

The tool consisted of four subscales: (1) personalized stigma, (2) disclosure concerns, (3) negative self-image, and (4) public attitudes, of which each question can be rated based on a 4-point Likert scale (strongly disagree, disagree, agree, and strongly agree).^21,22^ The third section was related to HRQoL comprising the Medical Outcomes Study HIV Health Survey (MOS-HIV), HRQoL measurement tool specifically developed for HIV-infected individuals, and the EuroQoL 5-Dimension 5-Level (EQ-5D-5L) by the EuroQoL Group, the most widely used “multi-attribute utility” generic instrument for measuring HRQoL in cost-effectiveness studies.^23^ The MOS-HIV Health Survey was previously translated and validated in Thai PLWHIV by Chariyalertsak et al. (Cronbach's *α* > 0.70, except for the physical functioning subscale at Cronbach's *α* = 0.67).^24^

### Participant recruitment and data collection

A cross-sectional study was conducted in Phrao district, Chiang Mai province, of which data collection was carried out from July to October 2021. PLWHIV were eligible to be enrolled into the study if they were registered patients at Phrao hospital, at least 18 years of age or older, able to read and write in Thai, and willing to provide written informed consent for participation. The study was approved by the Research Ethics Committee, Faculty of Public Health, Chiang Mai University (Document No. ET012/2021). All participants provided written informed consent before their participation in the study.

Phrao hospital is a 60-bed district hospital that is responsible for health care of over 50,000 population in Phrao, a rural district 97 kilometers away from Chiang Mai city, the largest province in Northern Thailand. This community hospital has been involved in HIV/AIDS treatment and prevention for over three decades with established ARV clinic and “Malison” (Jasmine) self-help group serving as a platform for knowledge sharing and social support among PLWHIV.

Participants were convenient sample. The study team worked with the hospital's ARV clinic staff and Malison group leaders in reaching for the study participants. HIV patients followed up at the clinic were listed according to their residences in 11 subdistricts (Wiang, Thung Luang, Pa Tum, Pa Nai, San Sai, Ban Pong, Nam Phrae, Khuean Phak, Mae Waen, Mae Pang, and Long Khot). Participants were initially approached by ARV clinic staff or Malison group leaders. Then appointments for small group of 10–15 individuals, who were interested to participate, were made at one of Malison group leaders' residence or subdistrict hospital nearby their homes.

At the beginning of each session, the study objectives, informed consent, and study questionnaire were briefly presented. After consent, all participants were allowed to complete the questionnaires themselves privately, while the study research team was available if they needed assistance or clarifications. It took ∼15–30 min for survey completion and each participant was compensated with 100 Thai baht (approximately US$3) for their time.

The questions regarding ARV regimen, CD4 level, and viral load were put in a separate section and later completed by Phrao hospital ARV clinic staff based on the hospital medical record. Study data were initially collected in paper, and then entered and managed using REDCap electronic data capture tools hosted at the Research Institute for Health Sciences, Chiang Mai University, a secure, web-based application designed to support data capture for research studies.^25^

### Data analysis

The data entered were checked and verified before exporting into Stata/IC version 16.0 for Windows (StataCorp LP, College Station, TX, USA) for analyses. Descriptive statistics, including frequency, percentage, mean, standard deviation (SD), and minimum and maximum values, were conducted to analyze participants' demographic and clinical characteristics.

Factors related to stigma, and HRQoL were investigated using linear regression analysis. Variables of interest, including sex, age, marital status, educational level, occupation, socioeconomic status, residence, duration since HIV diagnosis, CD4 cell count, viral load, ART duration, and regimen, were selected in the univariable analysis, of which those with *p* value <0.20 were carried out in the multi-variable analysis with a statistically significant level of 0.05. Pearson correlation was used to test the correlations between stigma and quality–of-life variables, with the significant level set at *α* = 0.05. There was no evidence indicating multi-collinearity between the independent variables (all VIFs were close to 1).^26^ All significant levels reported are two sided with *p* value <0.05.

## Results

### Respondent characteristics

From ∼300 PLWHIV in Phrao district, we could reach 165 potential participants, of which 161 eligible individuals voluntarily participated in the study (97.6%). The respondents were from all 11 subdistricts, of which 74 were males and 87 were females (Table 1). The mean age was ∼50 years (range, 18–76 years). More than half of respondents were married (*n* = 88, 54.7%). Over one-fourth were separated, divorced, or widowed (*n* = 45, 27.9%), while less than one-fifth were single (*n* = 28, 17.4%). Most participants completed primary school (81.4%), while only 16 and 2% completed high school or vocational certificate, and bachelor's degree or higher, respectively.

**Table 1.** Respondent Demographics (*N* = 161)

Characteristics	*n* (%)
Sex	
Male	74 (46.0)
Female	87 (54.0)
Age (years)	
≤40	16 (9.9)
41–50	57 (35.4)
51–60	66 (41.0)
>60	22 (13.7)
Mean ± SD	51.1 ± 9.0
Min–max	18–76
Marital status	
Single	28 (17.4)
Married/living with a partner	88 (54.7)
Divorced/separated/widowed	45 (27.9)
Educational level	
Primary school	131 (81.4)
High school/vocational or high vocational certificate	26 (16.1)
Bachelor's degree or higher	4 (2.5)
Occupation	
Unemployed	13 (8.1)
Employed	148 (91.9)
Average monthly income (THB^a^ ± SD)	4501 ± 3341
Government officials/private sector employee	4 (2.5)
Average monthly income (THB^a^ ± SD)	13,570 ± 5132.2
Min–max	7000–18,000
Free-lance/merchant	103 (64.0)
Average monthly income (THB^a^ ± SD)	4296 ± 3112.3
Min–max	500–20,000
Agriculture	41 (25.5)
Average monthly income (THB^a^ ± SD)	4110 ± 2363.2
Min–max	300–9000
Average number of days missed from work during the past 3 months due to illness (day ± SD)	3 ± 4.4
Socioeconomic status	
Having financial difficulty	77 (47.8)
Sufficient money for living	84 (52.2)
Residence	
Own house	116 (72.0)
Children or relative's house	37 (23.0)
Others	8 (5.0)
Health insurance	
UHC	147 (91.3)
Social security scheme	9 (5.6)
CSMBS	2 (1.2)
Others, for example, children or partners' health insurance	3 (1.9)

^a^ The average market exchange rate in Q3, 2021 was $US1 = 32.92 THB.^27^

CSMBS, Civil Servant Medical Benefit Scheme; SD, standard deviation; THB, Thai Baht; UHC, universal health coverage.

Most of the sample were employed (92%), in which majority were freelancers or merchants (64%), followed by farmers (25.5%). The average monthly income was 4500 baht (US$ 137). Those working in agriculture sector were among the lowest income strata. The average number of days missed from work due to illness over the past 3 months was 3 days. About half reported having sufficient money for living (52.2%), whereas nearly half (47.8%) had financial difficulty. Nearly one-fourth (72%) lived in their own residence. Most of respondents were covered by universal health coverage scheme (UCS) for their medical expenses.

### HIV-related variables

The mean duration since HIV diagnosis was 15 years ranging between 1 month and 35 years (SD = 6.0). All participants received ART with an average duration of 13 years (SD = 4.8). Nucleoside/nucleotide reverse transcriptase inhibitors (NRTIs) in combination with a non-nucleoside reverse transcriptase inhibitor were the most common regimen used (88.8%), whereas NRTIs plus a protease inhibitor (PI) were taken among 18 participants (11.2%). The mean CD4 cell count was 736.6 cells/mm^3^ (SD = 327.4). Most patients had plasma viral load less than 20 copies/mL (Table 2).

**Table 2.** HIV-Related Variables (*N* = 161)

HIV-related variables	*n* (%)
Duration since HIV diagnosis (years)	
Mean ± SD	15.2 ± 6.0
Min–max	0.1–35
Duration received ART (years)	
Mean ± SD	13.0 ± 4.8
Min–max	0.1–21
ART type	
2NRTIs + NNRTI	143 (88.8)
TDF +3TC or FTC + EFV	108 (75.5)
TDF +3TC or FTC + NVP	7 (4.9)
AZT +3TC or FTC + EFV	11 (7.7)
AZT +3TC + NVP	8 (5.6)
Other^a^	9 (6.3)
2NRTIs + PI	18 (11.2)
TDF +3TC + LPV/RTV	10 (55.6)
AZT + TDF + LPV/RTV	5 (27.8)
Other^b^	3 (16.6)
CD4 cell count (cells/mm^3^)	
<500	36 (22.4)
≥500	124 (77.0)
Missing data^c^	1 (0.6)
Mean ± SD	736.6 ± 327.4
Min – max	140–1989
CD4 cell count (%)	
<25	77 (47.9)
≥25	82 (50.9)
Missing data^c^	2 (1.2)
Mean ± SD	25.75 ± 7.78
Min – max	9.2–51.2
Viral load (copied/mL)	
<20	147 (91.3)
20–100	11 (6.9)
>100	2 (1.2)
Missing data^c^	1 (0.6)

^a^ Include ABC +3TC + EFV, ABC +3TC + NVP, AZT +3TC + RPV.

^b^ Include AZT +3TC + LPV/r, 3TC + EFV + LPV/RTV.

^c^ No patient history data from the hospital database system.

AZT, Zidovudine; EFV, Efavirenz; FTC, Emtricitabine; LPV, Lopinavir; NNRTIs, non-nucleoside reverse transcriptase inhibitors; NRTIs, nucleoside/nucleotide reverse transcriptase inhibitors; NVP, Nevirapine; PIs, protease inhibitors; RTV, Ritonavir; SD, standard deviation; 3TC, Lamivudine; TDF, Tenofovir Disoproxil Fumarate.

### HIV stigma and HRQoL

The overall HIV stigma score based on the 12-item stigma scale was 28.31 (SD = 5.43). Negative self-image had the highest mean score (7.37 points, SD = 1.57), followed by disclosure concerns (7.17 points, SD = 1.87), personalized stigma (7.00 points, SD = 1.51), and public attitudes (6.77 points, SD = 1.60), respectively (Table 3).

**Table 3.** Respondent's HIV Stigma and Health-Related Quality of Life Score (*N* = 161)

Variables	Mean ± SD	Min–max
HIV stigma		
Overall	28.31 ± 5.43	14–45
Personalized stigma	7.00 ± 1.51	3–12
Disclosure concerns	7.17 ± 1.87	3–12
Negative self-image	7.37 ± 1.57	3–12
Public attitudes	6.77 ± 1.60	3–11
MOS-HIV		
PHS	49.43 ± 9.08	17.01–63.07
MHS	48.46 ± 8.65	16.87–66.13
General health perceptions	48.01 ± 18.52	5–100
Pain	65.08 ± 19.99	11–100
Physical functioning	79.40 ± 24.17	0–100
Role functioning	83.23 ± 33.05	0–100
Social functioning	79.50 ± 23.12	0–100
Mental health	67.08 ± 16.73	12–100
Vitality/energy	61.21 ± 18.29	5–100
Health distress	73.17 ± 20.35	0–100
Cognitive functioning	78.45 ± 17.51	15–100
Quality of life	65.84 ± 20.29	0–100
Health transition	57.30 ± 21.04	0–100
EQ-5D-5L		
Health utility	0.912 ± 0.149	−0.056 to 1.000
VAS score	75.84 ± 17.06	10–100

EQ-5D-5L, EuroQoL 5-Dimension 5-Level; MHS, mental health summary; MOS-HIV, Medical Outcomes Study HIV Health Survey; PHS, physical health summary; SD, standard deviation; VAS, visual analog scale.

According to the MOS-HIV questionnaire, the mean physical health summary (PHS) score was 49.43 and the mental health summary (MHS) score was 48.46. The highest mean was found in the role functioning subscale (83.23 points), while the lowest mean was found in the general health perceptions subscale (48.01 points) (Table 3). In terms of general HRQoL, the mean health utility (HU) was 0.91 based on EQ-5D-5L, while the mean self-rated score measured with the visual analog scale (VAS) for overall health was 75.8 (Table 3). About half of respondents had problems with pain and discomfort (55.9%), while over one-third had issues concerning anxiety/depression (38.5%). However, the severity degree was mostly mild to moderate.

### Factors associated with HIV stigma and HRQoL

The multi-variable analysis to examine factors related to HIV stigma and QoL was conducted (Table 4). The model revealed that marital status and duration of ART were significantly associated with the HIV stigma (*R*^2^ = 11.4%). Married people had 2.61 lower stigma score than single/divorced/separated/widowed people (*p* = 0.002), while each one more year of ART use was associated with declining HIV-related stigma score by 0.27 (*p* = 0.002).

**Table 4.** Factors Associated with HIV Stigma and Health-Related Quality of Life (*N* = 161)

Outcome	Factor	Coef.	SE	*p*	95% CI	VIF
HIV stigma	Marital status	−2.610	0.814	0.002	−4.218 to −1.003	1.00
	Duration of ART	−0.271	0.085	0.002	−0.440 to −0.102	1.00
	Constant	33.269	1.265	<0.001	30.770 to 35.768	
PHS	Age	−1.809	0.774	0.021	−3.337 to −0.281	1.13
	Occupation	6.930	2.298	0.003	2.391 to 11.468	1.04
	Socioeconomic status	4.898	1.326	<0.001	2.278 to 7.518	1.16
	Residence	4.714	1.411	0.001	1.926 to 7.501	1.06
	Constant	35.077	3.362	<0.001	28.436 to 41.719	
MHS	Age	−1.902	0.724	0.009	−3.332 to −0.473	1.11
	Occupation	8.583	2.182	<0.001	4.272 to 12.894	1.05
	Socioeconomic status	2.681	1.234	0.031	0.243 to 5.118	1.13
	HIV stigma	−0.535	0.109	<0.001	−0.750 to −0.319	1.04
	Constant	54.638	4.525	<0.001	45.698 to 63.577	
Health utility	Age	−0.027	0.013	0.031	−0.052 to −0.003	1.11
	Occupation	0.205	0.037	<0.001	0.131 to 0.279	1.04
	Socioeconomic status	0.066	0.021	0.002	0.024 to 0.108	1.12
	ART regimen	−0.064	0.032	0.049	−0.127 to 0.000	1.02
	Constant	0.738	0.064	<0.001	0.611 to 0.865	
VAS score	Age	−3.561	1.392	0.011	−6.311 to −0.812	1.14
	Occupation	12.032	4.167	0.004	3.801 to 20.264	1.06
	Socioeconomic status	10.435	2.401	<0.001	5.692 to 15.178	1.19
	Residence	8.449	2.531	0.001	3.450 to 13.449	1.06
	Duration diagnosed with HIV	−0.379	0.190	0.048	−0.755 to −0.003	1.05
	HIV stigma	−0.505	0.210	0.017	−0.920 to −0.090	1.07
	Constant	68.504	9.477	<0.001	49.782 to 87.226	

Factor: age (≤40 years = 0, 41–50 years = 1, 51–60 years = 2, >60 years = 3); Marital status (single/divorced/separated/widowed = 0, married/living with a partner = 1); Occupation (unemployed = 0, employed = 1); Socioeconomic status (having financial difficulty = 0, sufficient money for living = 1); Residence (children or relative's house/others = 0, own house = 1); smoking (no = 0, yes = 1); ART type (2NRTIs + NNRTI = 0, 2NRTIs + PI = 1); Duration diagnosed with HIV, duration of ART, HIV stigma, PHS, MHS, health utility, and health-rated score = continuous data.

95% CI, 95% confidence interval; Coef., regression coefficient; MHS, mental health summary; PHS, physical health summary; SE, standard error; VAS, visual analog scale; VIF, variance inflation factor.

Age, occupation, and socioeconomic status were significant predictors associated with all HRQoL measures, of which increase in age was inversely correlated with HRQoL, whereas the others showed positive correlations. Living in own residence (*p* = 0.001) and higher HIV stigma score (*p* < 0.001) were additional positive and negative predictors for PHS and MHS score, respectively.

Advanced age (*p* = 0.031) and receiving 2NRTI + PI were negatively correlated with HU (*p* = 0.049), while being employed (*p* < 0.001) and having sufficient money for living (*p* = 0.002) were associated with higher level of HU. After stratifying by sex, males who were married showed higher HU by 0.076 compared to their single, divorced/separated counterparts (*p* = 0.036). However, this relationship was not significant among females.

Greater age (*p* = 0.011), duration diagnosed with HIV (*p* = 0.048), and HIV stigma (*p* = 0.017) were negatively associated with VAS score, while being employed (*p* = 0.004), having sufficient money for living (*p* < 0.001), and living in own residence (*p* = 0.001) were associated with higher VAS score.

### Relationships between stigma and HRQoL

The correlation coefficients among HIV-related stigma, PHS, MHS, HU, and VAS are presented in Table 5. Significant negative correlations were observed between HIV stigma and MHS (*r* = −0.351, *p* < 0.001), while significant positive correlations were observed between PHS and MHS (*r* = 0.671, *p* < 0.001), PHS and HU (*r* = 0.619, *p* < 0.001), PHS and VAS score (*r* = 0.738, *p* < 0.001), MHS and HU (*r* = 0.504, *p* < 0.001), MHS and VAS score (*r* = 0.609, *p* < 0.001), and HU and VAS score (*r* = 0.538, *p* < 0.001).

**Table 5.** Pearson Correlation Coefficient (*r*) Between HIV Stigma and Health-Related Quality of Life (*N* = 161)

Variable	1	2	3	4	5
1. HIV stigma	1				
2. PHS	−0.113	1			
3. MHS	−0.351^**^	0.671^**^	1		
4. HU	−0.034	0.619^**^	0.504^**^	1	
5. VAS score	−0.137	0.738^**^	0.609^**^	0.538^**^	1

^**^ *p* < 0.01.

HU, health utility; MHS, mental health summary; PHS, physical health summary; VAS, visual analog scale.

## Discussion

This cross-sectional study identified multiple factors associated with HIV-related stigma and discrimination and HRQoL among PLWHIV in rural Thailand setting and their integrated contribution. Marital status (being married/living with a partner compared to being single/divorced/separated/widowed) and duration of ART were negatively correlated with HIV stigma, while increase in age was inversely associated with HRQoL (PHS, MHS, HU, and VAS score). On the other hand, being employed and socioeconomic status (having sufficient money for living compared to having financial difficulty) positively predicted HRQoL, with living in own residence as an additional positive predictor for PHS and VAS score. HIV stigma was negatively associated with MHS and VAS score, whereas duration diagnosed with HIV and using 2NNRTIs in combination with PI regimen were negative factors associated with VAS and HU, respectively. The main findings are in line with previous work that social factors and financial security have impact on stigma and individual resilience.^28–31^

Being married/living with a partner and duration of ART were associated with a decline in HIV stigma. This could explain that marital status is beneficial in terms of perceived social and psychological support, while PLWHIV who have been using ART for a long time may have better acceptance and adjusted their day to day living tasks better compared to newly diagnosed individuals who are treatment naive. A correlational study in Thai women showed that marital status had significant influence on personalized stigma, public attitude, and negative self-image.^32^ The fact that 2NRTIs in combination with NNRI was the common regimen used, despite the long treatment duration, and most participants have CD4 over 500 cells/mm^3^ with less than 20 copies/mL viral load imply that they had good compliance with low rate of ARV resistance.

Being married or living with a partner was predictor associated to the improvement in HRQoL, while after sex-stratifying analysis, married men appeared to have higher level of HU score compared to their single peers. However, this relationship was not significant among women. The results are consistent with previous findings,^33–35^ with more disruption in HRQoL overtime among married women.^35^ This may suggest that marriage may provide more benefits to men than women as the demand of marital relationship, household responsibilities, and caring for children may exhaust women and create greater pressure.

Both socioeconomic status and employment correlate positively to better HRQoL. However, being employed appears to have stronger association than socioeconomic status. This is plausible because employment status can capture better the socioeconomic determinants and HRQoL in other dimensions, including daily living, a network of social support and identity.^34^

In this study, negative self-image subscale score was the highest, followed by disclosure concerns, which is in line with the study among Thai youth living with HIV.^21^ Internalized stigma has been noted as more important and impactful than external stigma and discrimination.^36^ A cross-sectional study in the Netherlands demonstrated the mediating effect of self-stigma on both perceived public stigma and experienced stigma on quality-of-life outcomes.^37^ This is probable that “perceived beliefs” are potentially more likely to initiate feelings or fear of being stigmatized rather than the actual stigma experiences.^37^

Currently, the data regarding effectiveness of stigma intervention, particularly internalized stigma, are limited. Effectiveness of structural-level interventions, including provision of ART, economic and social empowerment, as well as individual-level cognitive behavioral therapy in reducing HIV self-stigma, was observed in low- and middle-income countries.^38^ Therefore, efforts to reduce internalized stigma among PLWHIV at individual level with specific interventions to foster their disclosure and contentious attitudes toward the infection would be paramount.

The significant relationships between HIV stigma and HRQoL variables reflect their connections. Higher level of stigma shows negative impact on mental health, while PHS, MHS, HU, and VAS are all positively correlated. This is consistent with observed associations between stigma and HRQoL found in previous studies.^28,39^ This emphasizes the complex relationship of the issues that PLWHIV are facing and the need to address all components together holistically.

The study provides additional insights regarding the contribution to HIV-related stigma and HRQoL prediction under PLWHIV's perspective in rural Thailand context. The questionnaire used comprehensively involved standard validated tools, including the abbreviated 12-item stigma scale, the MOS-HIV, and the EQ-5D-5L. The validated 12-item short version of stigma scale used has facilitated the completion, but still preserves the main elements and broad concepts of the original 40-item version.^22^ To our knowledge, our study is the first study using the 12-item stigma scale in Thai population after its validation and reported consistent results concerning HIV stigma scores with the previous work.^21^

Both generic and HIV-specific tools were employed for HRQoL measurement according to the recommendation from previous reviews.^40,41^ The MOS-HIV was one of the instruments with most established psychometric properties for HIV-specific measure, while the selected generic measure, EQ-5D-DL, can serve as useful adjunct to the MOS-HIV. The use of self-administered questionnaire should have facilitated respondents' privacy and some comfort to answer questions more straight forwardly. However, there are notable limitations. The fact that the data were collected in Phrao district, Chiang Mai, Thailand, which is a single study in rural setting, may limit the generalizability of results in other settings. Our study population is relatively older compared to the country's estimate and previous studies.^24,42^ This was probably because younger patients and adolescents are reluctant to disclose their status and less likely to participate in the study.

However, the marital and socioeconomic status and ART regimen use are in line with previous studies.^24,33,42^ The nature of cross-sectional design used prevented us to explore causal relationship of the identified associations. In addition, due to the limited sample, gender differences in key population (including MSM, TGW, and SWs) were not examined. The use of convenience sample and self-reported measure might have potentially missed some patients with higher levels of stigma or poorer outcomes. Notwithstanding these limitations, all clinical variables obtained were from the hospital medical record, indicating the reliability of data. More research under PLWHIV's perspective to address these limitations as well as development of effective interventions targeting at individual level are warranted.

This cross-sectional study identifies predictors associated with HIV-related stigma (marital status and duration of ART) and HRQoL (age, occupation, and socioeconomic status) and their relationships under PLWHIV's perspective. HIV-related stigma remains a significant challenge that affects HRQoL and other social aspects. The design of effective intervention targeted at individual will close the gap.
